# Endometriosis Is Associated with a Significant Increase in hTERC and Altered Telomere/Telomerase Associated Genes in the Eutopic Endometrium, an Ex-Vivo and In Silico Study

**DOI:** 10.3390/biomedicines8120588

**Published:** 2020-12-09

**Authors:** Rafah Alnafakh, Fiona Choi, Alice Bradfield, Meera Adishesh, Gabriele Saretzki, Dharani K. Hapangama

**Affiliations:** 1 Department of Women’s and Children’s Health, Institute of Life Course and Medical Sciences, University of Liverpool, Member of Liverpool Health Partners, Liverpool L8 7SS, UK; R.A.A.Alnafakh@liverpool.ac.uk (R.A.); F.Choi2@student.liverpool.ac.uk (F.C.); a.j.bradfield@liverpool.ac.uk (A.B.); meeadish@liverpool.ac.uk (M.A.); 2 Department of Pathology, Al-Hilla Teaching Hospital, Babil 51001, Iraq; 3 Liverpool Women’s Hospital NHS Foundation Trust, Member of Liverpool Health Partners, Liverpool L8 7SS, UK; 4 Biosciences Institute, Campus for Ageing and Vitality, University of Newcastle, Newcastle upon Tyne NE4 5PL, UK; gabriele.saretzki@newcastle.ac.uk

**Keywords:** hTERC, *DKC1*, dyskerin, telomere, telomerase, endometriosis

## Abstract

Telomeres protect chromosomal ends and they are maintained by the specialised enzyme, telomerase. Endometriosis is a common gynaecological disease and high telomerase activity and higher hTERT levels associated with longer endometrial telomere lengths are characteristics of eutopic secretory endometrial aberrations of women with endometriosis. Our ex-vivo study examined the levels of hTERC and DKC1 RNA and dyskerin protein levels in the endometrium from healthy women and those with endometriosis (*n* = 117). The in silico study examined endometriosis-specific telomere- and telomerase-associated gene (TTAG) transcriptional aberrations of secretory phase eutopic endometrium utilising publicly available microarray datasets. Eutopic secretory endometrial hTERC levels were significantly increased in women with endometriosis compared to healthy endometrium, yet dyskerin mRNA and protein levels were unperturbed. Our in silico study identified 10 TTAGs (*CDKN2A, PML, ZNHIT2, UBE3A, MCCC2, HSPC159, FGFR2, PIK3C2A, RALGAPA1,* and *HNRNPA2B1*) to be altered in mid-secretory endometrium of women with endometriosis. High levels of hTERC and the identified other TTAGs might be part of the established alteration in the eutopic endometrial telomerase biology in women with endometriosis in the secretory phase of the endometrium and our data informs future research to unravel the fundamental involvement of telomerase in the pathogenesis of endometriosis.

## 1. Introduction

Telomeres are specialised nucleoprotein complexes at the chromosomal ends containing repeated nucleotide sequences ((TTAGGG)n) and shelterin proteins [1]. They protect the genome from nucleolytic degradation, undesired recombination, repair, and end-to-end chromosome fusion [2] while they are maintained by a specialised enzyme, telomerase. The human telomerase enzyme is composed of 3 core subunits: (1) The telomerase RNA component (hTERC), (2) the catalytic subunit telomerase reverse transcriptase (hTERT) and (3) the dyskerin protein [3].

Telomerase activity (TA) shows dynamic changes in human endometrium correlating with the ovarian cycle and with glandular proliferation [4]. Within the endometrium, stromal cells, regardless of the cycle phase, possess very low levels or absent TA and hTERC in comparison with epithelial cells [4,5,6,7]. Proliferating endometrial epithelial cells have the highest amount of TA [4] and it was suggested that the elevated TA in epithelial cells could preferentially cap and maintain their short telomeres in order to protect endometrial epithelial cell telomeres from shortening to a critical length [4]. Direct in vitro inhibition of TA using the TERC inhibitor “imetelstat” resulted in inhibition of cell proliferation and prevention of gland formation in healthy human endometrial epithelial cells [4]. The relatively quiescent postmenopausal endometrium shows low levels of TA [8].

Endometriosis is a common gynaecological disease defined as the presence of endometrial glands and stroma-like lesions outside the uterus and it is an oestrogen-dependent disease, which can cause significant morbidity in a large number of women of reproductive age [9,10]. Unfortunately, there are no curative treatments and none of the available treatments is effective in managing the symptoms associated with this disease [11]. The pathogenesis of endometriosis is not yet fully elucidated in order to develop new treatments for endometriosis-associated pain and infertility. Therefore, women with endometriosis continue to suffer and they have a decreased quality of life [11].

The best-accepted theory of the pathogenesis of endometriosis is retrograde menstruation, by seeding of eutopic endometrium in the pelvis that initiates endometriotic deposits [12]. However, this phenomenon of retrograde menstruation occurs in almost all women [13] while the prevalence of endometriosis in the general population is only 10–15% of women. Thus, this theory had subsequently been modified to propose that eutopic endometrium shed by women with endometriosis to have particular aberrations, enhancing its ectopic growth [14,15]. Supporting this theory, there is a plethora of evidence confirming that the endometrium of women with endometriosis differs from that of healthy fertile women [16,17]. High TA levels, higher *hTERT* gene expression and hTERT protein levels associated with longer mean endometrial telomere length (TL) have all been reported to be characteristics of such abnormalities specific to eutopic secretory endometrium of women with endometriosis when compared with the endometrium of healthy women [7,14,18,19,20,21].

During retrograde menstruation, elevated TA in late-secretory endometrium of women with endometriosis [19] may cause the sloughed cells to survive in the peritoneal cavity and establish ectopic lesions [4,14]. While TA, hTERT, and TL have been studied in the context of endometriosis; there have not yet been any reports published on the levels of the remaining core components, hTERC and dyskerin, in the endometrium of women with endometriosis to date. 

Therefore, our aim was initially to examine the levels of hTERC and DKC1 RNA as well as dyskerin protein levels in normal endometrial tissue obtained from healthy women and in endometria collected from those with a surgical diagnosis of benign proliferative endometrial disease, endometriosis. We then progressed to an in silico study, where endometriosis-specific telomere- and telomerase-associated transcriptional aberrations of secretory phase eutopic endometrium were explored. Using a comprehensive telomere- and telomerase-associated genes (TTAGs) list that was compiled in our previous work [22], differentially regulated TTAGs were examined in published human endometrial microarray datasets from women with and without endometriosis, which were selected according to a strict predetermined criteria.

## 2. Experimental Section

### 2.1. Study Group

Full-thickness and pipelle biopsies were collected from healthy (endometriosis surgically excluded) pre and postmenopausal women undergoing hysterectomy at the Liverpool Women’s Hospital (LWH). Eutopic endometrial samples were collected from women with and without endometriosis during the secretory phase as well as the ectopic endometriotic samples that were surgically excised from women with peritoneal endometriosis as part of their surgical treatment during the period 2009 to 2017. From hysterectomy specimens, a wedge of tissue from the lumen to the muscular myometrial layer that included superficial and basal endometrium as well as myometrium was taken. A pipelle endometrial sampler was used to sample the endometrial functionalis layer of women undergoing laparoscopy. The collection of human tissue was approved by the Liverpool Adult Research Ethics Committee (LREC 09/H1005/55, 19/SC/0449) and informed written consent was obtained from all patients. Study groups included healthy proliferative phase (PP) (*n* = 27), secretory phase (SP) (*n* = 23), and normal postmenopausal (PM) samples (*n* = 33). Additionally, 34 samples were collected from women with endometriosis which included secretory phase eutopic endometrium (*n* = 19) and ectopic lesions (*n* = 15) (not matched) (Table 1). Tissue samples were collected as, (1) fixed (≥24 h in 4% (*v*/*v*) buffered formalin) and paraffin-embedded for immunohistochemical (IHC) staining, (2) some immediately placed into RNAlater (Sigma, Dorset, UK) for RNA extraction and qRT-PCR and (3) some snap-frozen immediately and kept in −80 °C for Telomeric Repeat Amplification Protocol (TRAP) assay (*n* = 6 for each of the different group studied). Additionally, myometrium underlying the endometrium were also collected from hysterectomy samples of premenopausal women (*n* = 4), and a fourth portion of the freshly harvested endometrial samples from 3 women were used for isolation of human endometrial epithelial and stromal fractions as previously described. Briefly, freshly-harvested endometrial tissue samples were mechanically and enzymatically digested to produce single cell suspensions. EpCAM (epithelial cell adhesion molecule) microbeads (Miltenyi Biotec Ltd., Surrey, UK) were used to positively select the freshly isolated epithelial cells according to the manufacturer’s protocol, while stromal fraction was isolated from EpCAM-depleted cells using selective adhesion. The purity of the sorted cell fractions was assessed by performing immunoblotting [4] and immunofluorescence for the expression of cytokeratin and vimentin Figure S1A,B. Additionally, cells maintained in culture were evaluated morphologically Figure S1C,D and by flow cytometry for the expression of glandular epithelial and stromal markers, CD9 and CD13 respectively, Figure S1E,F [4,21]. 

### 2.2. RNA Extraction and RT-qPCR 

RNA from frozen human endometrial and myometrial tissue preserved in RNA*later* (Thermo Fisher Scientific, Loughborough, UK) was extracted, quantified and reverse transcribed as previously described [17]. cDNA was amplified using iTaq universal SYBR Green supermix and CFX Connect Real-Time System (Bio-Rad, Hertfordshire, UK). HEK293 and Endometrial cancer cell line (Ishikawa) cDNA samples are the positive control used throughout RT-qPCR experiment for *DKC1* and hTERC respectively. The primer sequences and reaction conditions are listed in Table 2 [23,24,25,26,27]. For each target and reference, a standard curve was produced, and efficiency was examined (Figure S2A). The amplification products were run on an agarose gel (Figure S2B) to verify the specificity of the primers and to exclude any off-targets. The 2^−ΔΔCt^ method was used to calculate relative transcript level. *DKC1* was normalised to the geometric mean of peptidylprolyl Isomerase A *(PPIA*) [28] and tyrosine 3-monooxygenase/tryptophan 5-monooxygenase activation protein zeta (*YWHAZ*) [29], while *TERC* was normalised to the geometric mean of *PPIA* and beta-actin (*ACTB*) [28,29] using Bio-Rad CFX Manager software (Bio-Rad, Hertfordshire, UK).

### 2.3. Immunohistochemistry (IHC) Staining and Analysis

Standard IHC was performed on 3 μm serial sections of formalin fixed paraffin embedded (FFPE) endometrial tissue employing heat-induced antigen retrieval by pressure cooking in citrate buffer pH 6 for 3 min, and ImmPRESS polymerised peroxidase-based detection system (Vector Laboratories, Peterborough, UK) [30]. Anti-dyskerin primary antibody (Santa Cruz biotechnology, Texas, USA) was used at the concentration of (1:500). The primary antibody was incubated at 4 °C overnight and matching isotype control (rabbit IgG) (0.5 μg/mL) replaced the primary Ab as a negative control. A specific endometrial tissue sample with positive staining was included as an internal positive control with each staining experiment. Human tonsillar tissue was used as an external positive control.

The nuclear dyskerin immunoreactivity in both epithelial and stromal cells, in all relevant tissue samples, was quantified using a modified quickscore [17] by scanning the whole section and estimating the percentage of stained proportions in each of four intensities (0 = no staining, 1 = weak staining, 2 = moderate staining and 3 = strong staining). The total score out of 12 was calculated by multiplying the proportion of dyskerin positively stained cells (1–25% = 1, 26–50% = 2, 51–75% = 3 or >75 = 4) by the staining intensity score as previously described [17]. The staining was scored separately for in the cellular sub-compartments, i.e., luminal epithelium, stratum functionalis and basalis of premenopausal (healthy/endometriosis) as well as stratum basalis of normal postmenopausal endometrium, as well as epithelial and stromal cells, comprise of the ectopic endometriotic lesions. Two independent observers scored samples and discrepancies between their scores were resolved by re-evaluating the samples together and agreeing on a final score.

### 2.4. Telomeric Repeat Amplification Protocol (TRAP) Assay 

TA was measured using TeloTAGGG™ Telomerase PCR ELISA kit (Sigma-Aldrich, Dorset, UK) according to the manufacturers’ manual and as previously described [4], 1 µg of lysate was used. PCR was performed for 30 cycles using the conditions in accordance with the manufacturers’ manual. Optical density (OD) was measured at 450 nm in a Fluostar Omega Plate reader (BMG LABTECH, Aylesbury, UK) and presented as arbitrary units (AU). 

### 2.5. Statistical Analysis of the Ex-Vivo Study

Statistical differences between groups were calculated by non-parametric tests (Kruskal–Wallis or Mann–Whitney U-test) using Statistical Package for the Social Sciences (SPSS) version 25 (IBM Corp, Armonk, NY, USA). Graphs were plotted using GraphPad Prism 5 (GraphPad Software, La Jolla California USA). The correlation between immunostaining scores was determined with a Spearman test. *p*-value < 0.05 was considered to be significant.

### 2.6. In Silico Analysis of Endometrial Datasets and Identification of Differentially Regulated Genes in Mid-Secretory Phases

We searched the GEO database for available studies activating the following filters [31]: “Endometriosis” (study keyword), “datasets” AND “series” (Entry Type), “Homo sapiens” (organism), “Expression profiling by array” (study type). The two datasets GSE51981 and GSE6364 were chosen after applying strict selection criteria (Table 3) [32,33]. The array data for GSE51981 includes a total of 148 samples: 77 disease samples (28 mid-secretory) and 71 normal samples (22 mid-secretory); 14 “normal” samples were excluded as they contained other unspecified uterine pathologies. The array data for GSE6364 includes 37 samples: 21 diseased samples (9 mid-secretory) and 16 normal samples (8 mid-secretory). The web tool GEO2R (http://www.ncbi.nlm.nih.gov/geo/geo2r/) was used to identify differentially expressed genes (DEGs), between mid-secretory endometriosis samples and mid-secretory healthy controls. p < 0.05 and a |log2FC≥ 1| were set as the cut-off criteria. The DEGs were then filtered to include only TTAGs, using a list compiled in our previous work [22]. Volcano plots of the DEGs were produced using the web application VolcaNoseR (https://huygens.science.uva.nl/VolcaNoseR/). Following this, DEGs that were common to both datasets were determined and venn diagrams were constructed using the Bioinformatics and Evolutionary Genomics web-tool (http://bioinformatics.psb.ugent.be/webtools/Venn/). Functional analysis of DEGs was performed using Enrichr (https://amp.pharm.mssm.edu/Enrichr/) via Gene Ontology (GO) and Kyoto Encyclopedia of Genes and Genomes (KEGG) enrichment analyses [34,35]. *p* < 0.01 was chosen as the cut-off. The web tool REVIGO (http://revigo.irb.hr/) was then utilised to condense the list of biological process GO terms into a smaller representative list by removing similar GO terms [36]. Similarity was set at < 0.05.

### 2.7. Identification of Key Transcription Factors 

Once up- and downregulated TTAG genes were determined, they were entered into oPOSSUM 3.0 (http://www.cisreg.ca/oPOSSUM/) to identify enriched potential transcription factor (TF) binding sites (TFBS) in their promoter regions. Human Single Site Analysis (SSA) was implemented on vertebrate-specific TFBS; all genes were compared against all 24,752 genes in the oPOSSUM database. A list of relevant TFs regulating the genes close to the promoter region was obtained by using 2000/0 on the upstream/downstream score. A conservation cut-off was set at 0.60 and a matrix threshold at 80%. TFs were analysed by Fisher scores; the score compares the proportion of a set of genes containing a particular TFBS motif to the proportion of the background set that contains the motif [37]. The top ten TFBSs were sorted by Fisher’s score.

## 3. Results

### 3.1. Telomerase Holoenzyme Component (hTERC and DKC1) Levels Demonstrate a Dynamic Expression Pattern in Healthy Endometrium

We examined the dynamic changes across the menstrual cycle in normal premenopausal endometrium and in the PM endometrium, by using qPCR for hTERC and DKC1 RNA levels, IHC for dyskerin protein levels and the TRAP assay for TA levels. 

#### 3.1.1. hTERC and DKC1 RNA

**Figure 1:**
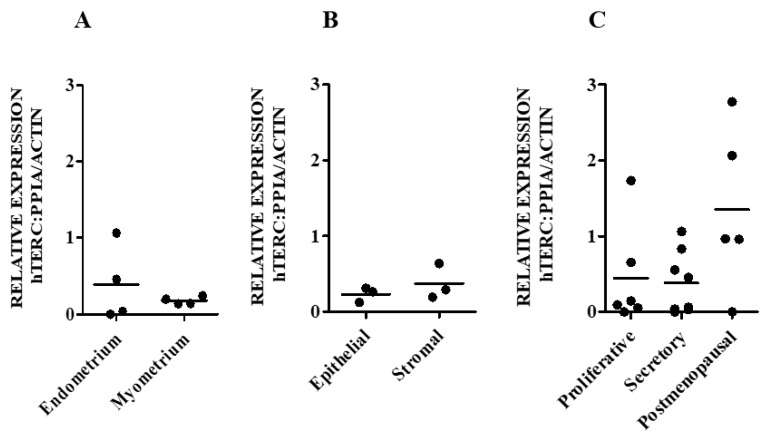
hTERC RNA levels in normal human uterus. hTERC RNA levels were measured using qPCR in the following samples: (**A**) Endometrium (E) (*n* = 4) versus myometrium (M) (*n* = 4). (**B**) Isolated epithelial (Ep) (*n* = 3) versus stromal (St) (*n* = 3) endmetrial cells. (**C**) Proliferative (PP) (*n* = 6), secretory phase (SP) (*n* = 8) and PM (*n* = 5) endometrial samples. *hTERC* expression was normalised to the geometric mean of peptidylprolyl Isomerase A (*PPIA*) and beta-actin (*ACTB*) reference genes.

**Figure 2:**
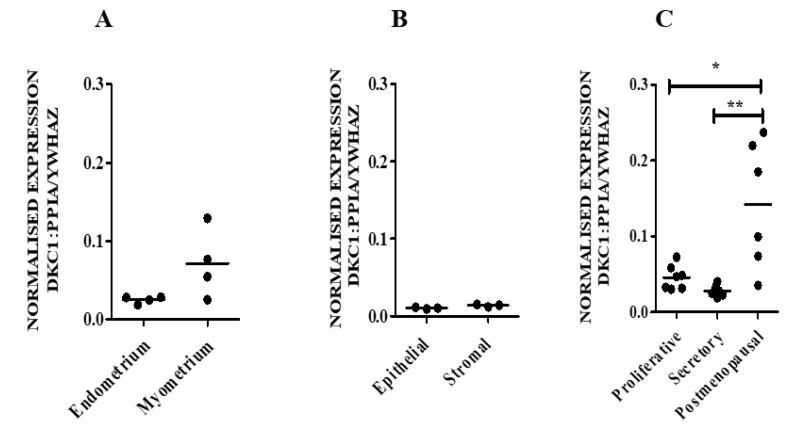
DKC1 mRNA levels in normal human uterus. DKC1 mRNA was measured in the following endometrial samples: (**A**) Endometrium E (*n* = 4) versus myometrium (M) (*n* = 4). (**B**) Isolated epithelial (Ep) (*n* = 3) versus stromal (St) (*n* = 3) endometrial cells. (**C**) Proliferative (PP) (*n* = 7), secretory phase (SP) (*n* = 8) and postmenopausal (PM) (*n* = 6) endometrial samples, *DKC1* expression was normalised to the geometric mean of peptidylprolyl Isomerase A (*PPIA*) and tyrosine 3-monooxygenase/tryptophan 5-monooxygenase activation protein zeta (*YWHAZ*) reference genes, * *p* < 0.05, ** *p* < 0.01.

hTERC and DKC1 RNAs are present in both endometrial and myometrial samples (Figure 1A and Figure 2A) at apparently similar levels. Likewise, hTERC and DKC1 RNA levels appeared to be similar in freshly sorted endometrial epithelial and stromal cells (*p* = 0.7 and *p* = 0.1 respectively) (Figure 1B and Figure 2B). Healthy postmenopausal endometrium appeared to have higher levels of hTERC RNA (*p* = 0.1) (Figure 1C) and significantly higher DKC1 mRNA compared with normal premenopausal endometrial samples (postmenopausal vs proliferative *p* = 0.01; postmenopausal vs secretory phase, *p* = 0.001; Figure 2C). 

There were no significant differences observed in hTERC or DKC1 RNA levels between the proliferative and secretory phase samples from healthy premenopausal women (Figure 1C and Figure 2C). In healthy human pre and postmenopausal endometrial samples, there was a trend of a positive correlation between DKC1 and hTERC RNA levels (Spearman co-efficient r = 0.44).

#### 3.1.2. Dyskerin Protein

**Figure 3:**
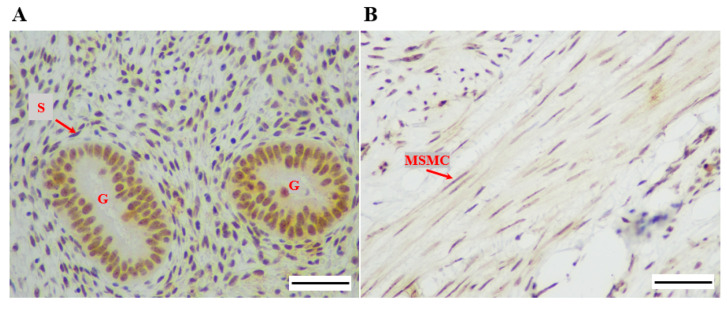
Microphotographs demonstrating dyskerin immunostaining at the cellular level in (**A**) human endometrium (proliferative phase), (**B**) myometrium. Magnification 400×. Scale bar 50 μm. Glands (G), stromal cells (S), myometrial smooth muscle cell (MSMC).

IHC staining revealed the presence of dyskerin protein and immunostaining was pri-marily localised in the nucleus and/or nucleolus in the endometrium (Figure 3A) and myometrium (Figure 3B). 

**Figure 4:**
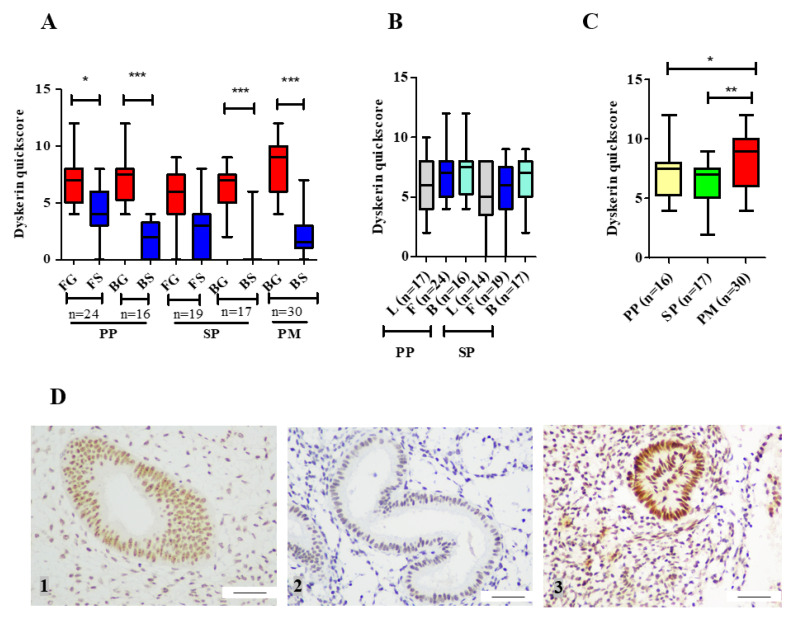
Dyskerin immunostaining in human endometrial tissue samples. (**A**) The difference in dyskerin immunoscores between epithelial and stromal compartments in functionalis and basalis layers of proliferative phase (PP), secretory phase (SP), and postmenopausal (PM) endometrium. Functionalis gland (FG), Functionalis stroma (FS), basalis gland (BG), and basalis stroma (BS). (**B**) Dyskerin immunoscores in the epithelium of the three endometrial layers: Luminal (L), functionalis (F) and basalis (B) of premenopausal PP and SP endometrium. (**C**) Dyskerin quickscores in basalis glands of PP (*n* = 16), SP (*n* = 17) and PM endometria (*n* = 30). (**D**) Representative microphotographs illustrating dyskerin immunostaining in the following endometrial samples (1) healthy proliferative phase (PP), (2) Secretory phase (SP) and (3) Healthy postmenopausal (PM) endometrium. Positive staining appears brown. Magnification 400×. Scale bar 50 μm. (BS). * *p* < 0.05, ** *p* < 0.01, *** *p* < 0.001.

In both functionalis and basalis layers, epithelial cells consistently demonstrated stronger dyskerin immunoreactivity than the stromal cells (*p* < 0.001, Figure 4A), when the individual cell types (epithelium and stroma) were considered, no region-specific differences were observed (Figure 4A,B). Dyskerin immunoscores were significantly higher in healthy postmenopausal endometrial epithelium compared with the basalis of premenopausal endometrial samples endometria (*p*=0.001). This difference was significant when samples of proliferative or secretory phase were compared with postmenopausal endometrium (proliferative vs postmenopausal, *p* = 0.03; secretory vs postmenopausal, *p* = 0.002; Figure 4C). Figure 4D shows the immunostaining in proliferative, secretory and postmenopausal endometrium. 

**Figure 5:**
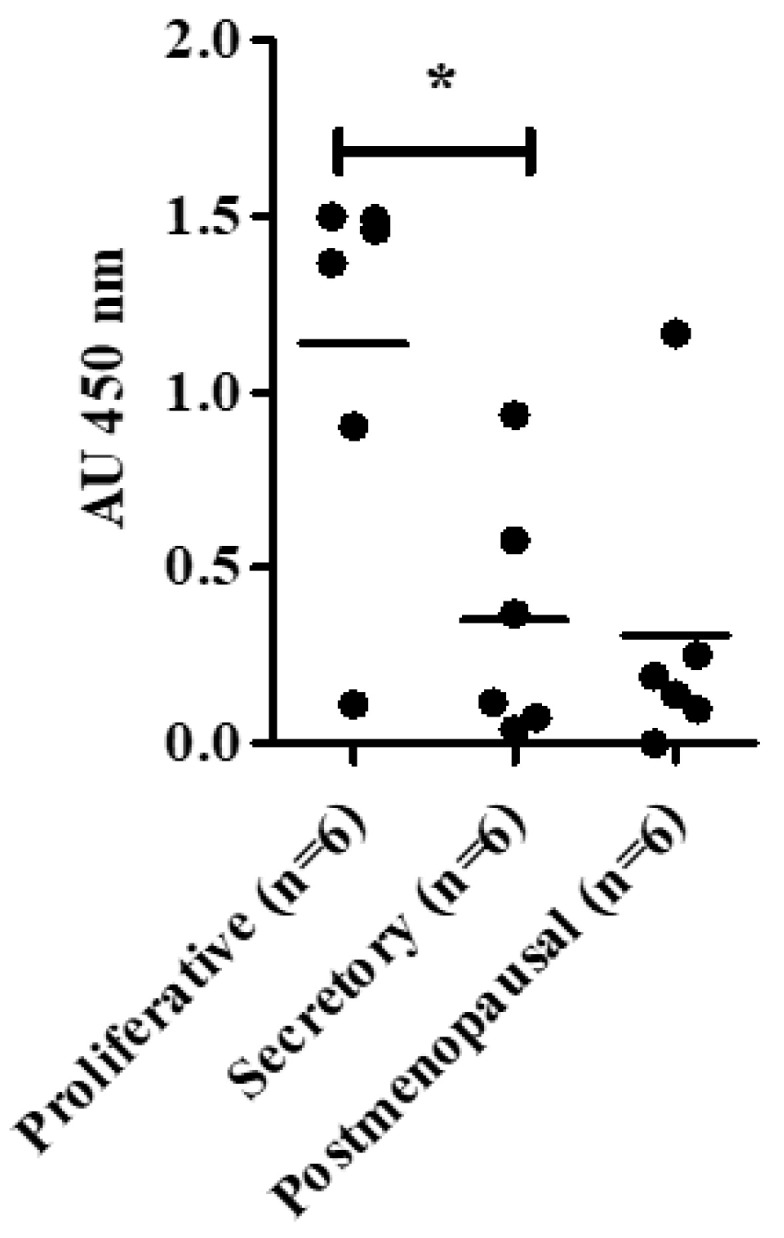
Telomerase activity (TA) in human endometrial tissue samples. TA was measured using Telomerase Repeat Amplification Protocol (TRAP) assay. Proliferative (PP) (*n* = 6), secretory phase (SP) (*n* = 6) and postmenopausal (PM) endometrial samples (*n* = 6). * *p* < 0.05.

Our sample cohort demonstrated well an established pattern of TA in proliferative phase samples demonstrating significantly higher levels in comparison to the secretory phase (*p* = 0.04) (Figure 5). TA in the proliferative endometrium also appeared to be higher compared with healthy postmenopausal endometrium (*p* = 0.1) (Figure 5).

### 3.2. hTERC Levels Were Significantly Increased Without Affecting Dyskerin (DKC1) Levels in the Secretory Eutopic Endometrium of Women with Endometriosis

Lowest TA in the normal endometrium has been reported in the secretory phase of the premenopausal endometrium and the most prominent endometrial differences in women with endometriosis are observed in the secretory phase of the cycle [4]. Furthermore, the secretory endometrium of women with endometriosis had been reported to have significantly higher TA compared with normal healthy endometrium [18]. Considering all these previous data, we selected secretory phase endometrium of women with and without endometriosis to examine endometriosis associated differences in hTERC and DKC1 RNA levels using qPCR and dyskerin protein with IHC.

**Figure 6:**
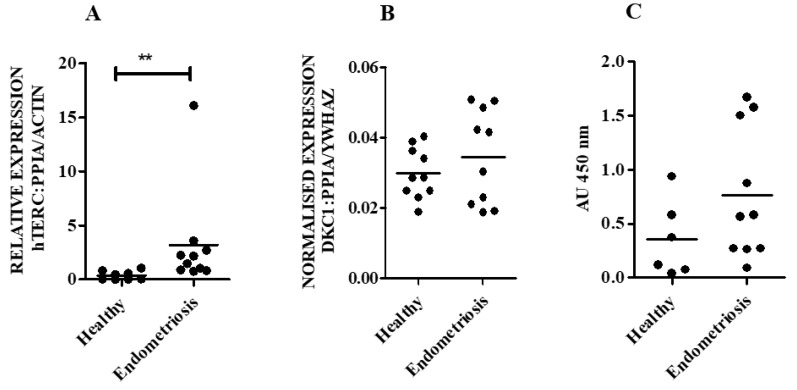
Eutopic secretory phase (SP) endometrium from women with confirmed endometriosis compared with SP from healthy women. (**A**) Human hTERC RNA in healthy secretory phase (SP) (*n* = 8) and secretory phase from women with endometriosis (*n* = 10). The hTERC RNA was normalised to the geometric mean of peptidylprolyl Isomerase A (PPIA) and beta actin (ACTB) reference genes. (**B**) DKC1 mRNA in healthy SP (*n* = 10) and eutopic SP from women with endometriosis (*n* = 10). The DKC1 mRNA was normalised to the geometric mean of PPIA and tyrosine 3-monooxygenase/tryptophan 5-monooxygenase activation protein zeta (YWHAZ) reference genes. (**C**) Telomerase activity (TA) in healthy SP (*n* = 6) and in SP from women with endometriosis (*n* = 10). ** *p* < 0.01.

hTERC RNA levels were significantly higher in eutopic secretory endometrium from patients with endometriosis, when compared with healthy secretory endometrial samples (*p* = 0.001) (Figure 6A). DKC1 mRNA levels and TA appeared to be higher in the endometrium of women with endometriosis compared with those from healthy women (*p* = 0.5) but the difference did not reach statistical significance (Figure 6B,C). No correlation was observed between hTERC RNA and TA in a group of healthy or endometriosis secretory endometrial samples (Spearman co-efficient r = 0.−20, r = 0.03 respectively).

**Figure 7:**
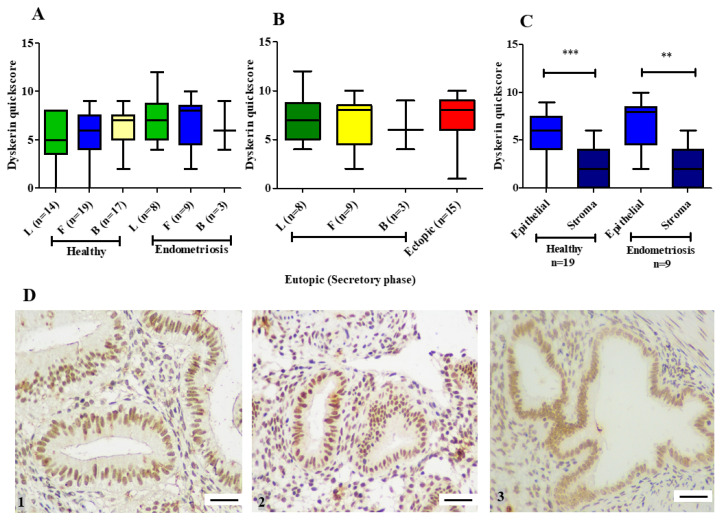
Dyskerin immunostaining in the healthy secretory phase, eutopic (secretory phase) endometrium from patients with confirmed endometriosis and ectopic endometriotic tissue (**A**) Dyskerin immunoscores in the three endometrial layers luminal (L), functionalis (F) and basalis (B) of healthy secretory phase compared with corresponding layers of eutopic endometrial samples from patients with endometriosis. (**B**) Dyskerin immunoscores comparison of L (*n* = 8), F (*n* = 9) and B (*n* = 3) endometrial layers from eutopic secretory phase (SP) endometriotic samples with ectopic endometriotic lesions (*n* = 15). (**C**) Dyskerin immunoscores in functionalis glands (FG) and stroma (FS) of SP samples from healthy women (*n* = 19) and from women with confirmed endometriosis (*n* = 9) ** *p* < 0.01, *** *p* < 0.001. (**D**) Representative microphotographs show dyskerin immunostaining in healthy endometrial secretory phase (1), eutopic endometrium from a patient with endometriosis (2) and ectopic endometriotic tissue (3). Positive staining appears brown. Magnification 400×. Scale bar 50 μm. ** *p* < 0.01, *** *p* < 0.001.

Dyskerin immunoreactivity in all three layers of secretory eutopic endometrium (luminal, functionalis, and basalis) of women with endometriosis were compared with the corresponding layers of the endometrium from healthy premenopausal women in the secretory phase (Figure 7A). Dyskerin immunoscores showed a trend towards being higher without statistical significance in the secretory luminal and functionalis epithelial regions of women with endometriosis (Figure 7A). There was no significant difference between the dyskerin scores of ectopic endometriotic epithelium (Figure 7B) when compared with all 3 layers of eutopic endometrium (luminal, functionalis, and basalis epithelium) of women with endometriosis (*p* = 0.5, *p* = 0.8, *p* = 0.4 respectively) (Figure 7B). Stromal dyskerin immunoscores were similar in the endometrium collected from both healthy and endometriosis groups of women (Figure 6C). Consistent with the observation in the healthy endometrium, in the endometrium of women with endometriosis, epithelial dyskerin scores were consistently higher than the stroma (Figure 7C). Figure 7D shows dyskerin immunohistochemistry staining in samples from women with endometriosis (eutopic and ectopic) as well as in secretory eutopic endometrium collected from a healthy woman. 

### 3.3. In Silico Interrogation of Publicly Available Datasets Demonstrates other Telomerase Associated Genes to Be Altered in the Mid-Secretory Endometrium of Women with Endometriosis

We then wanted to examine the aberrations in telomerase- and telomere-associated gene (TTAG) expression in the endometrium in women with endometriosis, beyond the 2 telomerase components we examined. In order to do that, we selected published endometrial microarray datasets generated from women with endometriosis in the secretory phase and control datasets derived from secretory endometrial samples of healthy women. We also employed a comprehensive list of TTAGs that we had previously generated and published [22] and interrogated the above endometrial datasets to identify abnormally expressed TTAGs. 

**Figure 8:**
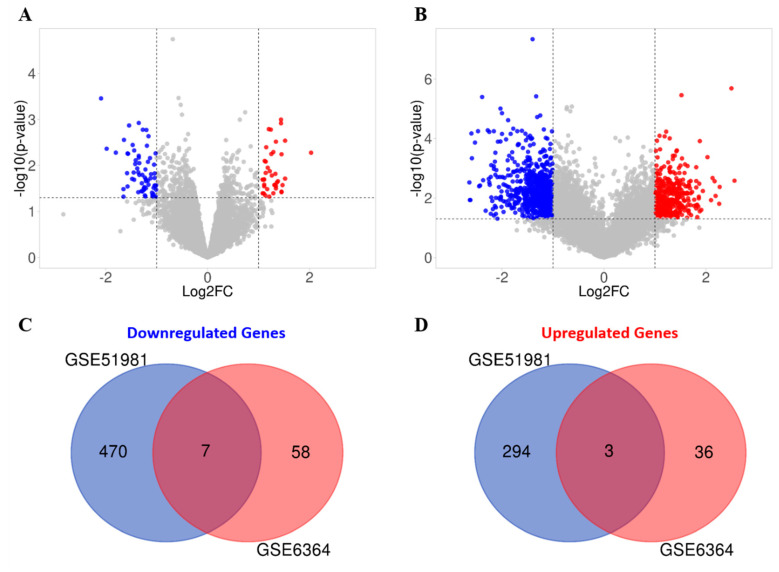
Differentially expressed genes (DEGs) in mid-secretory endometrial samples from women with endometriosis, compared with healthy controls from the (**A**) GSE6364 and (**B**) GSE51981 datasets, shown as Volcano plots. Cut-off criteria: |log2FC > 1| and *p* < 0.05. Red denotes upregulation, and blue denotes downregulation. (**C**) Venn diagram demonstrating upregulated genes in both GSE51981 (blue) and GSE6364 (red) and the common genes between both datasets (purple). (**D**) Venn diagram demonstrating downregulated genes in both GSE51981 (blue) and GSE6364 (red) and the common genes between both datasets (purple).

By applying aforementioned cut-off criteria and filtering of TTAGs from the methods section, and by comparing endometrial samples from women with endometriosis and those without endometriosis in the mid-secretory phase of the cycle, 104 (39 upregulated and 65 downregulated) and 774 (297 upregulated, 477 downregulated) DEGs were identified from GSE6364 and GSE51981 datasets, respectively (Figure 8A,B). Seven downregulated (Figure 8C) and three upregulated TTAGs (Figure 8D) were found to be common to both datasets (Table 4).

#### 3.3.1. Functional Enrichment Analysis 

Functional enrichment analysis was performed on the 10 common DEGs and revealed 4 KEGG pathways, 76 biological process GO terms, 10 molecular function GO terms and 1 cellular component GO term (Table 5). Due to the large number of biological process terms, the web tool REVIGO was utilised to condense this into a smaller representative list. After removing similar terms, 25 biological process GO terms remained. Biological process terms included “cellular senescence”, “positive regulation of telomere maintenance”, “gland morphogenesis”, and “progesterone receptor signalling pathway”. In molecular function analysis, the DEGs showed enrichment in Phosphatidylinositol kinase activity and telomeric DNA binding. The cellular component analysis revealed that the DEGs were predominantly located in the telomeric region of the chromosome.

#### 3.3.2. Identification of Key Transcription Factors 

Transcriptional gene regulation is dependent on transcription factors (TFs) that bind directly to promotor sites or regions of regulated genes [38]; hence, identifying TFBS for differentially expressed TTAGs provides additional information on gene regulation.Using oPOSSUM analysis we identified the top 10 enriched TFBS, ranked by Fisher Score, amongst the 10 commonly dysregulated TTAGs. These TFBS bind to the following TFs: SOX2, POU5F1, RORA_2, HNF1A, PPARG::RXRA, Spz1, MEF2A, MIZF, ESR2, and HIF1A::ARNT (Table 6).

## 4. Discussion 

Involvement of telomerase in endometrial biology is well established. Previous reports have demonstrated human endometrial epithelial proliferation to be associated with high TA and increased hTERT expression [4,8]. Consequently, increased TA and *hTERT* levels were observed in the endometrium of women with the proliferative endometrial disease, endometriosis [18,19]. Our ex-vivo study with patient-derived endometrial samples initially examines the involvement of the remaining two main core components of telomerase holoenzyme (hTERC and dyskerin) in healthy human endometrium across all stages of the premenopausal menstrual cycle, in postmenopausal endometrium as well as the involvement of these two components in eutopic endometrium of women with endometriosis. The dyskerin immunostaining of ectopic endometriotic lesions was also examined. Our data demonstrate dynamic changes in these components in pre/postmenopausal samples and reveals that eutopic secretory endometrial hTERC RNA levels are significantly increased in women with endometriosis. Contrastingly, dyskerin (DKC1) mRNA and protein levels were unperturbed in the secretory phase eutopic endometrium of women with endometriosis. Subsequently, we expanded this work with an in silico study, analysing available microarray datasets to determine the differentially regulated TTAGs in the mid-secretory endometrium of women with endometriosis to determine the involvement of these in known aberrations of telomerase biology and cellular function in the pathogenesis of endometriosis. Interestingly, *hTERT* was not upregulated in either of the datasets; this is contrary to prior findings that *hTERT* expression is higher in the eutopic secretory endometrium of women with endometriosis when compared with the endometrium of healthy women [7,14,18,19,20,21]. This is consistent with previous observations in endometrial cancer, a condition associated with high TA Even the large TCGA dataset did not show an increased expression of *hTERT* in women with endometrial cancer [22]. Consistent with experimental data, mid-secretory expression of *DKC1* in mid-secretory endometrium from women with endometriosis was not significantly different when compared with the control healthy samples according to published datasets. Although no expression data were available for *hTERC*, 10 additional TTAGs were found to be altered in mid-secretory endometrium of diseased patients (Figure 8). 

The current consensus is that all core components of the telomerase enzyme will contribute to TA [3]. The endometrial TA and hTERC RNA levels did not correlate in our study, and previous authors have reported that the enzymatic level of TA measured by TRAP assay, does not necessarily correlate with *hTERC* expression [39,40]. hTERC is ubiquitously expressed in all tissues, thus some have suggested hTERC levels to be not directly relevant to TA [39], therefore, supporting our observation in the endometrium. In vitro studies have suggested that hTERT needs hTERC as a minimal requisite for TA but that the main catalytic subunit hTERT might be the rate limiting feature relevant to TA [41]. In the human endometrium, epithelial TA and hTERT RNA levels have been relevant to cell proliferation and are regulated by ovarian steroid hormones, progesterone and estradiol [4,7]. Eutopic endometrium of women with endometriosis appears to be different to that of women without endometriosis and the aberration in the endometrial functionalis layer in these women have been proposed to play a role in the pathogenesis of endometriosis [7,9,14,15,42,43]. Previous studies have also demonstrated that TA, hTERT protein and mRNA levels were increased in the secretory phase endometrium of women with endometriosis [18,19]. The high TA accompanying the pro-proliferative, anti-apoptotic, and senescence-evading phenotype found in the secretory phase endometrium of women with endometriosis, has been proposed to contribute to endometriotic lesion formation due to deposition of sloughed endometrial fragments after retrograde menstruation [14,20]. Our study examined hTERC and *DKC1* expression levels in eutopic endometrium of women with endometriosis demonstrating significantly higher hTERC RNA in the secretory phase compared with healthy secretory endometrium. RNA interference of hTERC reduced cancer cell proliferation rate quickly, without altering TA [44]. We therefore hypothesise that the high hTERC RNA levels found in the eutopic endometrium of women with the benign proliferative disease, endometriosis, may facilitate the postulated increased proliferative potential of these cells, which may also be a reflection of increased TA, but dyskerin may not play a major role in the pathology of endometriosis.

Dyskerin is the main component of the dyskerin core complex (dyskerin-NHP2-NOP2) that binds and stabilises hTERC, and thus enhances TA. The expression levels of both genes are expected to follow a similar pattern to endometrial *hTERT* and TA. However, the regulation of the steady-state levels of hTERC is far more complex [45]. hTERC transcripts undergo a crucial multistep process of maturation, which collectively controls the levels of hTERC and dysregulation of these steps in various pathologies may influence TA. Functional TA in vivo, however, requires more than just hTERT and hTERC, but dyskerin and other associated components of the larger telomerase holoenzyme as well as some proteins that are only transiently associated with the holoenzyme [45].

There is previous data however, supporting an intricate relationship between hTERC and dyskerin, where a reduction in DKC1 mRNA or dyskerin protein was shown to be associated with a concomitant reduction in hTERC levels. For example, the A353V mutation in the *DKC1* gene, which is the most common mutation in X linked dyskeratosis congenita (X-DC) patients, causes a reduction in the levels of hTERC RNA [46], while another report revealed that loss of dyskerin caused degradation of hTERC in vivo [47]. In breast cancer tissue, when DKC1 mRNA levels were very low, TA was significantly decreased, independently of the level of *hTERT* expression [48]. Additionally, in vitro *DKC1* gene knock-down using siRNA in MCF-7 breast cancer cells, reduced TA via a reduction in hTERC levels [48]. Taken together, this evidence suggests the simultaneous reduction of DKC1 and hTERC was associated with a concomitant reduction in TA. In contrast, in our study we only found changes in hTERC RNA levels but not for dyskerin expression and protein levels. Unlike premenopausal endometrial TA levels, hTERC and DKC1 RNA levels do not demonstrate a significant change across the menstrual cycle. Furthermore, in complete contrast to TA, healthy premenopausal endometrial hTERC and DKC1 RNA levels were low in premenopausal endometrium while significantly increased levels were observed in proliferatively quiescent postmenopausal samples. When previous evidence is considered in this context, although knocking down *DKC1* influenced TA via hTERC in MCF-7 cells as mentioned above, an excessive reduction of hTERC levels was required to decrease TA [48]. Thus, we propose that the reduced levels seen in hTERC in premenopausal proliferative endometrium in the context of high TA levels, may still not have reached the threshold level to affect endometrial TA. In contrast, in the postmenopausal endometrium, where TA was low, DKC1 RNA level was high in comparison with premenopausal samples; therefore, we hypothesise that the high DKC1 levels could possibly have a direct consequence on endometrial TA. Since dyskerin plays an important role in stabilising hTERC, high levels of dyskerin protein may be important in resisting carcinogenesis in the postmenopausal endometrium via the non-telomerase related pathway. Dyskerin acts also as pseudouridine synthase which catalyses the isomerisation of uridine to pseudouridine in rRNA. *DKC1* mutations cause ribosomal dysfunction and result in a reduction of tumour suppressive proteins. For example, in a X-DC mouse model, defective translation of specific mRNAs harbouring internal ribosomal entry site (IRES) elements occurred due to decrease in dyskerin activity, and also reduced the tumour suppressor, p27, levels and caused a marked increase in spontaneous pituitary tumorigenesis in p27 heterozygous mice [49]. Further studies are therefore required to examine the functional consequence of over-expression of dyskerin, such as cellular proliferation.

Our novel finding that hTERC RNA levels are significantly higher in eutopic endometrial samples in the secretory phase of women with endometriosis, without altered levels of DKC1 mRNA, seems interesting. The high TA [19], hTERT [18,19], and hTERC (present study) levels may promote endometrial epithelial proliferation in women with endometriosis independent of dyskerin.

It is an established fact that not all excised endometriotic ectopic lesions contain endometrial epithelial and stromal-like cells are found during histological scrutiny. Techniques such as immunohistochemistry directly visualise the specimen to ascertain the cellular content, but other techniques such as PCR do not allow such assessment. Considering this discrepancy, we did not assess the RNA levels of hTERC or DKC1 in ectopic lesions. This inherent difficulty in ascertaining whether the excised presumed endometriotic lesions actually contained endometrium-like cells can be overcome in the future with employing specialist techniques such as in situ hybridisation.

By interrogating the published endometrial microarray datasets, we have identified 3 TTAGs that are specifically upregulated in the mid-secretory endometrium. *CDKN2A* being the most significantly upregulated (Table 4), is a known tumour suppressor gene, known to promote cell senescence [50]. The gene is known to decrease during cell proliferation therefore, our findings of CDKN2A upregulation corresponds to the expected halt in endometrial proliferation during the secretory phase [51]. Regardless, the loss of p16 has been described in many cancer entities [51] while in contrast, its high expression has been linked to poor prognosis of endometrial cancer, where telomerase and telomere activity are known to play an important role [52]. Moreover, there is a significant amount of evidence on altered p16 levels in eutopic endometrium in women with endometriosis [53]. Promyelocytic leukaemia (PML) is a critical component to the alternative telomere lengthening (ATL) pathway; its effects are achieved through ALT-associated PML bodies (APBs) [54]. APBs have recently been shown to drive BLM–TOP3A–RMI (BTR) accumulation which is known to promote break-induced replication-mediated telomere elongation [55]. Upregulation of PML may therefore suggest increased telomere-lengthening in endometriosis-positive mid-secretory endometrium. ZNHIT2 is a member of the zinc finger HIT domain-containing protein family and has not been well-characterised [56]. A recent study has demonstrated its role in regulating spliceosome activity [56]; spliceosome is a complex small nuclear RNA-protein machine responsible for removing introns from pre-mRNA; hence, known to regulate alternative splicing of a gene [57]. Therefore, altered ZNHIT2 levels seen in endometriosis may dysregulate alternative splicing of hTERT which is often found in cancers [58]. A total of 7 TTAGs were found to be commonly downregulated in both GEO datasets, suggesting that they may contribute to endometriosis pathogenesis. FGFR2 and PIK3C2A have both previously been proposed to contribute to endometriosis-related carcinogenesis [59,60], but their role in benign endometriosis has not been elucidated. PIK3C2A is associated with telomerase as a result of its yeast homologue, VPS34, being linked with telomere biology [61,62,63]. However, FGFR2 was thought to be associated with telomerase due to its downregulation in telomerase deficient cells in a previous study [63]. This is in contrast with our finding that FGFR2 was downregulated in the mid-secretory phase eutopic endometrium of women with endometriosis—a telomerase abundant tissue. FGFR2, which encodes fibroblast growth factor receptor 2, is involved in a wide array of cellular processes, such as cellular proliferation, differentiation, wound repair and regulation of tissue fibrosis [64]; its downregulation in endometriosis may be related to an alternative cellular process, irrespective of telomerase regulation. Both HNRNPA2B1 and HSPC159 have previously been linked with epithelial–mesenchymal transition (EMT) in cancer [65,66]; EMT is thought to drive malignant invasion and metastasis and has also been proposed to contribute, in part, to the detachment and dissemination of endometrial cells in endometriosis [67]. HNRNPA2B1 is linked with telomere biology as it has been shown to be upregulated upon telomere shortening in vitro [68] HSPC159 has been found in a fluorescence localisation screen to be located in close proximity to TINF2 [69], a protein from the shelterin protein complex that is bound to the telomere-binding proteins TRF1 and TRF2 [70]. The remaining identified genes, *UBE3A*, *MCCC2*, and *RALGAPA1*, have not previously been linked with endometrial disease, and very little is known about the actions of *MCCC2* and *RALGAPA1*. *UBE3A* is linked with telomere function as it has previously been shown to induce *hTERT* transcription and telomerase activity [71,72], whereas MCCC2 and RALGAPA1 have previously been found in a protein network surrounding TRF1, TRF2, and POT1 [73], which are all components of the shelterin complex [70]. A limitation to these in silico findings is that some of these TTAGs are only weakly associated with telomere function; thus, their dysregulation in endometriosis may be representative of other cellular functions. Our findings are also limited by the small sample size of the datasets. Nonetheless, we have identified multiple novel genes that potentially contribute to endometriosis and may warrant further study. Although the sample size is a major limitation of our ex-vivo study, we have selected a well characterised population of women at a specific time point in the cycle to reduce variability and identified a statistically significant difference in hTERC levels between women with and without endometriosis, which, in the context of prior data on endometrial telomerase and progesterone resistance, is biologically plausible. Future studies thus are warranted to further confirm this data by appropriately increasing the sample size. 

## 5. Conclusions

We have demonstrated for the first time high levels of hTERC RNA to be a part of the established alteration in the eutopic endometrial telomerase biology in women with endometriosis in the secretory phase of the endometrial cycle and our in silico work informs us of further avenues to explore in future studies to unravel the fundamental involvement of telomerase in the pathogenesis of endometriosis. 

## Figures and Tables

**Table 1:** Demographic features of study groups.

Study Groups	No.	* Age (years)	* BMI (kg /m^2^)
Proliferative phase	27	40(30–57)	27(18–41)
Secretory phase	23	41(21–48)	26(19–40)
Postmenopausal	33	63(40–85)	26(20–40)
Endometriosis	34	24(24–48)	24(11–35)

* Data expressed as median (range), body mass index (BMI).

**Table 2:** Primer sequences used for qPCR amplification.

Primer	Sequence	Amplicon	Annealing Temp (°C)	Efficiency (%)
*DKC1*	F:5’-CTCGGAAGTGGGGTTTAGGT-3	166	62	98
	R:5’-ACCACTTCAGCAACCACCTC-3			
*PPIA*	F:5’-AGACAAGGTCCCAAAGAC-3	118	60	100.10
	R:5’-ACCACCCTGACACATAAA-3			
*YWHAZ*	F:5’-CGTTACTTGGCTGAGGTTGCC-3	69	60	94.30
	R:5’-GTATGCTTGTTGTGACTGATCGAC-3			
*TERC*	F:5’-GCCTTCCACCGTTCATTCTA-3	220	60	86.1
	R:5’-CCTGAAAGGCCTGAACCTC-3			
*ACTB*	F:5’-TGTACGCCAACACAGTGCTG-3	183	60	94.6
	R:5’-GCTGGAAGGTGGACAGCGA-3			

**Table 3:** Inclusion and exclusion criteria to select microarray datasets for in silico analysis.

Inclusion	Exclusion
Phase-defined samples Human endometrium Patients diagnosed with endometriosis Patients free of hormonal treatment 3 months prior to biopsy Patients free of malignant/major systemic disease Study containing both diseased and normal tissue samples	Serum samples Non-endometrial tissue Epigenetic study carried out Primary cell culture of endometrial biopsies Study of non-human tissue Study of Malignancy Study of endometrial cell lines Study of adenomyosis Study of only diseased samples Samples that have not been cycle phase defined

**Table 4:** Common telomere- and telomerase-associated upregulated and downregulated genes in diseased mid-secretory phase endometrium compared with normal mid-secretory phase endometrium.

**Commonly Upregulated Genes**		
**Gene Symbol**	**Gene ID**	**Mean Log2FC**
*CDKN2A*	1029	1.255
*PML*	5371	1.295
*ZNHIT2*	741	1.315
**Commonly Downregulated Genes**		
**Gene Symbol**	**Gene ID**	**Mean Log2FC**
*UBE3A*	7337	−1.15
*MCCC2*	64087	−1.60
*HSPC159*	29094	−1.14
*FGFR2*	2263	−1.23
*PIK3C2A*	5286	−1.55
*RALGAPA1*	253959	−1.19
*HNRNPA2B1*	3181	−1.29

**Table 5:** Functional enrichment analysis of DEGs. Kyoto Gene and Genome Encyclopaedia (KEGG) pathways and GO terms were identified using Enrichr (*p* < 0.01). The list of biological process GO terms were condensed into a smaller representative list using REVIGO (similarity <0.5) and the top 10 biological process GO terms, according to *p*-value, are displayed.

	ID	Term	*p*-Value
KEGG	map05200	Pathways in cancer	0.001932257
	map04120	Ubiquitin mediated proteolysis	0.002022009
	map05203	Viral carcinogenesis	0.004288773
	map04144	Endocytosis	0.006253438
Biological Process (GO)	GO:0090398	Cellular senescence	6.17 × 10^–5^
	GO:200058	Regulation of protein ubiquitination involved in ubiquitin-dependent protein catabolic process	6.71 × 10^–5^
	GO:0032206	Positive regulation of telomere maintenance	1.11 × 10^–4^
	GO:0042325	Regulation of phosphorylation	0.001531221
	GO:0045926	Negative regulation of growth	0.001556759
	GO:2000109	Regulation of macrophage apoptotic process	0.002996578
	GO:0022612	Gland morphogenesis	0.002996578
	GO:0050657	Nucleic acid transport	0.003495224
	GO:0006551	Leucine metabolic process	0.003495224
	GO:0050847	Progesterone receptor signalling pathway	0.003495224
Molecular Function (GO)	GO:0016303	1-phosphatidylinositol-3-kinase activity	2.01 × 10^–4^
	GO:0052742	Phosphatidylinositol kinase activity	2.83 × 10^–4^
	GO:0035004	Phosphatidylinositol 3-kinase activity	6.29 × 10^–4^
	GO:0035005	1-phosphatidylinositol-4-phosphate 3-kinase activity	0.003495224
	GO:1990247	N6-methyladenosine-containing RNA binding	0.003495224
	GO:0098505	G-rich strand telomeric DNA binding	0.004989818
	GO:0004861	Cyclin-dependent protein serine/threonine kinase inhibitor activity	0.004989818
	GO:0032183	SUMO binding	0.005985094
	GO:0043047	Single-stranded telomeric DNA binding	0.005985094
	GO:0016307	Phosphatidylinositol phosphate kinase activity	0.007476328
Cellular Component (GO)	GO:0000781	Chromosome, telomeric region	0.00166095

**Table 6:** The top 10 transcription factors (TFs) whose binding sites were enriched in the telomere- and telomerase-associated DEGs, ranked by Fisher score. The TFBS were identified by oPOSSUM analysis.

Transcription Factor	Fisher Score
SOX2	5.904
POU5F1	5.826
RORA_2	4.746
HNF1A	4.476
PPARG::RXRA	4.455
Spz1	4.244
MEF2A	4.111
MIZF	3.906
ESR2	3.880
HIF1A::ARNT	3.805

## Supplementary Materials

The following are available online at https://www.mdpi.com/2227-9059/8/12/588/s1, Figure S1 Isolated endometrial cell characterization, Figure S2 RT-qPCR primers efficiency and specificity.
