# Supplementary material for: Endometriosis Is Associated with a Significant Increase in hTERC and Altered Telomere/Telomerase Associated Genes in the Eutopic Endometrium, an Ex-Vivo and In Silico Study
Source: Biomedicines. 2020 Dec 9;8(12):588. doi: 10.3390/biomedicines8120588 (PMC7764055; doi:10.3390/biomedicines8120588)
Supplement: Supplementary file 1 [file biomedicines-08-00588-s001.pdf]

Figure S1

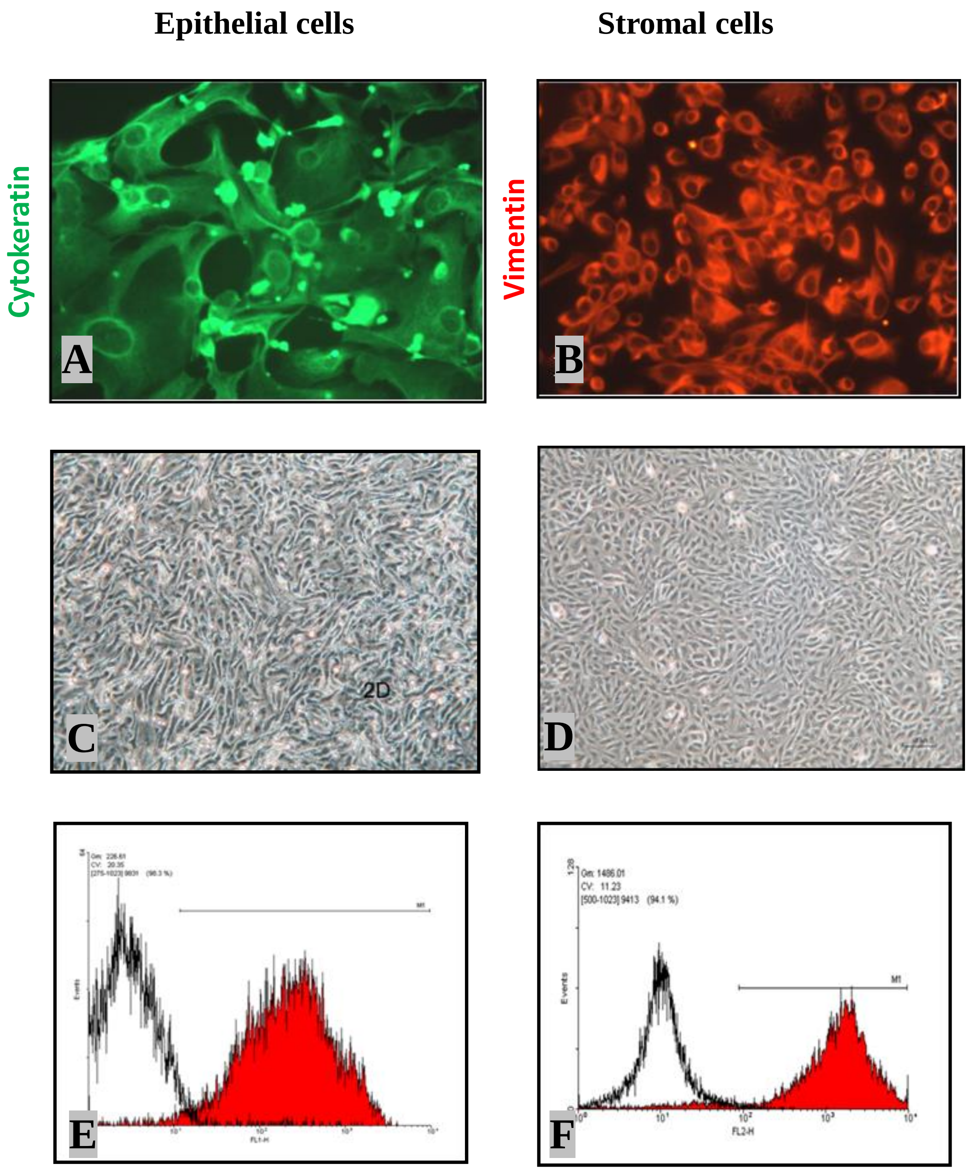

Epithelial cells
Stromal cells
Cytokeratin
Vimentin
A
B
2D
C
D
E
F

Figure S2

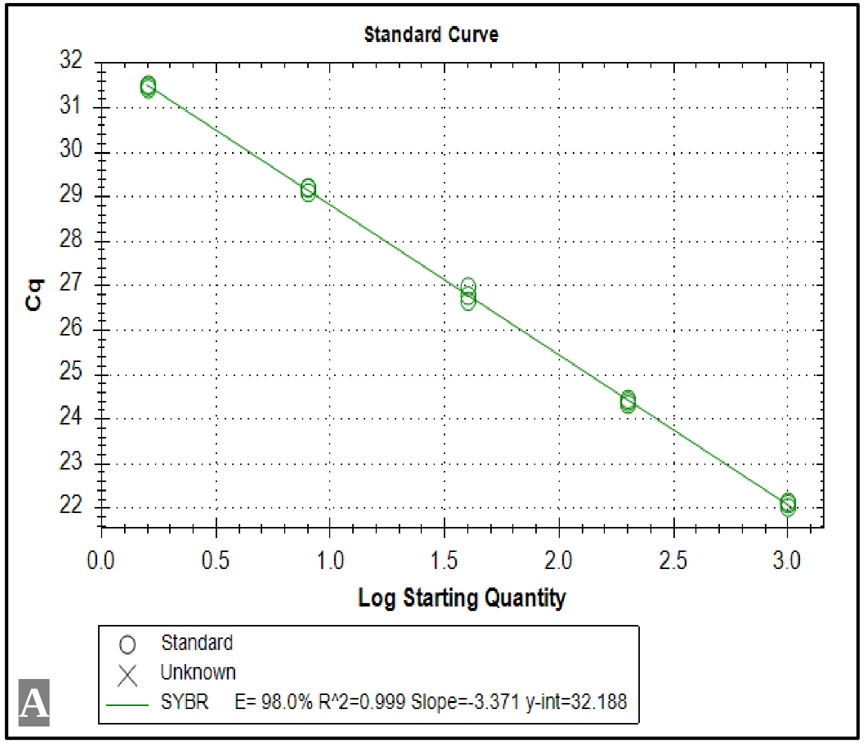
Standard Curve
Cq
32
31
30
29
28
27
26
25
24
23
22
0.0
0.5
1.0
1.5
2.0
2.5
3.0
Log Starting Quantity
Standard
Unknown
SYBR E= 98.0% R^2=0.999 Slope=-3.371 y-int=32.188
A

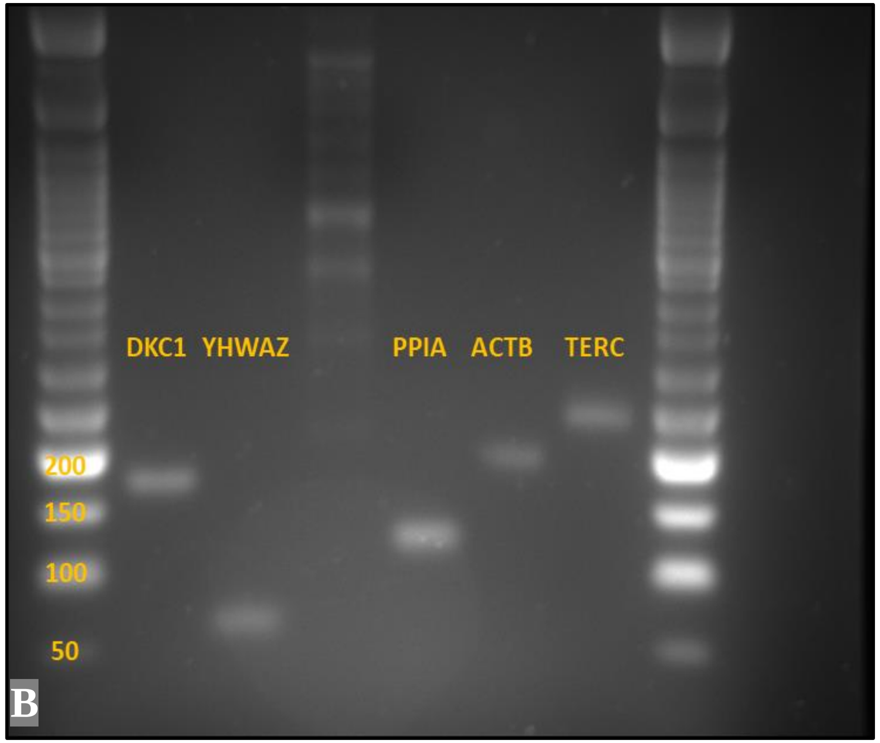
DKC1
YHWAZ
PPIA
ACTB
TERC
200
150
100
50
B

Figure S1 Isolated endometrial cell characterisation. Endometrial Biopsies were mechanically and enzymatically digested and purified using Epcam microbeads.

Epcam positive cells represent epithelial cells; Epcam negative cells are stromal cells. Purity of cultures was assessed by performing immunoblotting [4] and immunofluorescence for **A)** Cytokeratin and **B)**Vimentin. Purity of cultured cells also assessed morphologically by maintaining **C)** primary human epithelial and **B)** stromal cells in monolayer culture (2D), and by performing flow cytometry using **E)** Fluorescein conjugated antihuman CD9 and **F)** Phycoerythrin (PE) anti-human CD13. Representative histograms with red area representing labelled cells.

Figure S2 RT-qPCR primers efficiency and specificity A) DKC1 standard curve showing assay efficiency, precision and slop. Log starting quantity (nanogram). **B)** Image of agarose gel electrophoresis showing specific bands for DKC1, YHWAZ, PPIA, ACTB and hTERC.
